# Regional Cerebral Oxygen Saturation Monitoring in an Emergency Cesarean Section for Takayasu Arteritis: A Case Report on Utility and Limitations

**DOI:** 10.7759/cureus.105624

**Published:** 2026-03-21

**Authors:** Tokimitsu Hibino, Yusuke Okui, Yoshie Toba

**Affiliations:** 1 Department of Anesthesiology, Seirei Hamamatsu General Hospital, Hamamatsu, JPN

**Keywords:** common carotid artery stenosis, emergency cesarean section, numano classification, regional cerebral oxygen saturation (rso2), takayasu arteritis

## Abstract

Takayasu arteritis is a chronic inflammatory vasculitis primarily affecting the aorta and its major branches, such as the subclavian and common carotid arteries (CCAs). Because Takayasu arteritis predominantly affects young women of reproductive age, clinicians may encounter pregnant patients requiring complex perinatal management. In these patients, anesthesia for cesarean section is particularly challenging because peripheral blood pressure often fails to accurately reflect cerebral perfusion pressure due to extensive arterial stenosis. We report the anesthetic management of a 39-year-old pregnant woman with Takayasu arteritis and a history of a left parietal infarction ten years prior, who required an emergency cesarean section. Computed tomography at that time had already revealed severe, long-standing bilateral stenosis of the subclavian and CCAs; notably, the systolic blood pressure in her upper extremities was 60 mmHg lower than in her lower extremities upon admission for delivery. Given her compromised cerebral vascular reserve and the unreliability of peripheral blood pressure, we utilized regional cerebral oxygen saturation (rSO_2_) as the primary physiological indicator for cerebral perfusion. Although intense patient movement and sweating during labor initially destabilized the rSO_2_ sensors, measurements were successfully maintained by securing the sensors with additional adhesive tape and constant manual monitoring by dedicated staff. Under spinal anesthesia, we initiated vasopressor administration whenever rSO_2_ fell 20% below the baseline, a commonly used safety threshold for cerebral desaturation, preventing any subsequent decline of more than 30%. Both the mother and neonate were discharged without neurological sequelae. In patients with Takayasu arteritis and complex vascular lesions, rSO_2_ monitoring provides a critical real-time assessment of cerebral oxygenation when conventional blood pressure monitoring is unreliable. Clinicians must anticipate technical monitoring challenges in complex cases such as Takayasu arteritis during emergency deliveries and establish proactive backup plans to prevent perioperative neurological complications.

## Introduction

Takayasu arteritis (TA) is a chronic panarteritis causing progressive fibrosis and mural lesions, stenosis, occlusion, or dilatation, predominantly involving the aorta and its major branches in young women [1]. In Japan, its prevalence is approximately 40 cases per million [2]. Stenotic lesions frequently involve the subclavian and axillary (69%-71%), common carotid (37%-57%), and iliofemoral arteries (16%-20%) [3-5]. Hata et al. proposed the Numano classification based on an epidemiological study of TA patients in Japanese and Indian populations. This classification categorizes lesions based on their location: Ⅰ, aortic arch and its branches; Ⅱa, ascending aorta, aortic arch, and its branches; Ⅱb, Ⅱa lesions plus thoracic descending aorta; Ⅲ, thoracic descending aorta, abdominal aorta, renal arteries; IV, abdominal aorta and/or renal arteries; V, combination of IIb and IV (ascending aorta, aortic arch and its branches, thoracic descending aorta, plus abdominal aorta and/or renal arteries) [6]. Numano Type V, involving the entire aorta, accounts for nearly 49% of cases [3]. In such patients, limb-cuff blood pressure often underestimates cerebral perfusion pressure due to proximal arterial stenoses.

Although TA’s impact on fertility is debated, pregnancy after TA onset carries a 13-fold higher risk of obstetric complications (OR: 13; 95% CI: 5-33) [7-10]. Anesthetic management for patients with bilateral common carotid artery (CCA) stenosis is challenging because peripheral blood pressure measurements may not accurately reflect cerebral perfusion pressure. Here, we report an emergency cesarean section in a patient with TA and bilateral carotid stenosis. In such cases, severe stenosis of the carotid and subclavian arteries presents a unique challenge, as peripheral blood pressure measurements often fail to accurately reflect cerebral perfusion pressure. Near-infrared spectroscopy-derived regional cerebral oxygen saturation (rSO₂) monitoring serves as a non-invasive surrogate for cerebral blood flow, offering real-time insights into the balance between cerebral oxygen supply and demand. While its use in elective cases has been documented, this report focuses on the novel application of rSO₂ as the leading clinical threshold for hemodynamic management during an emergency cesarean section in a patient with TA, where conventional blood pressure monitoring was fundamentally limited [11].

## Case presentation

**Figure 1:**
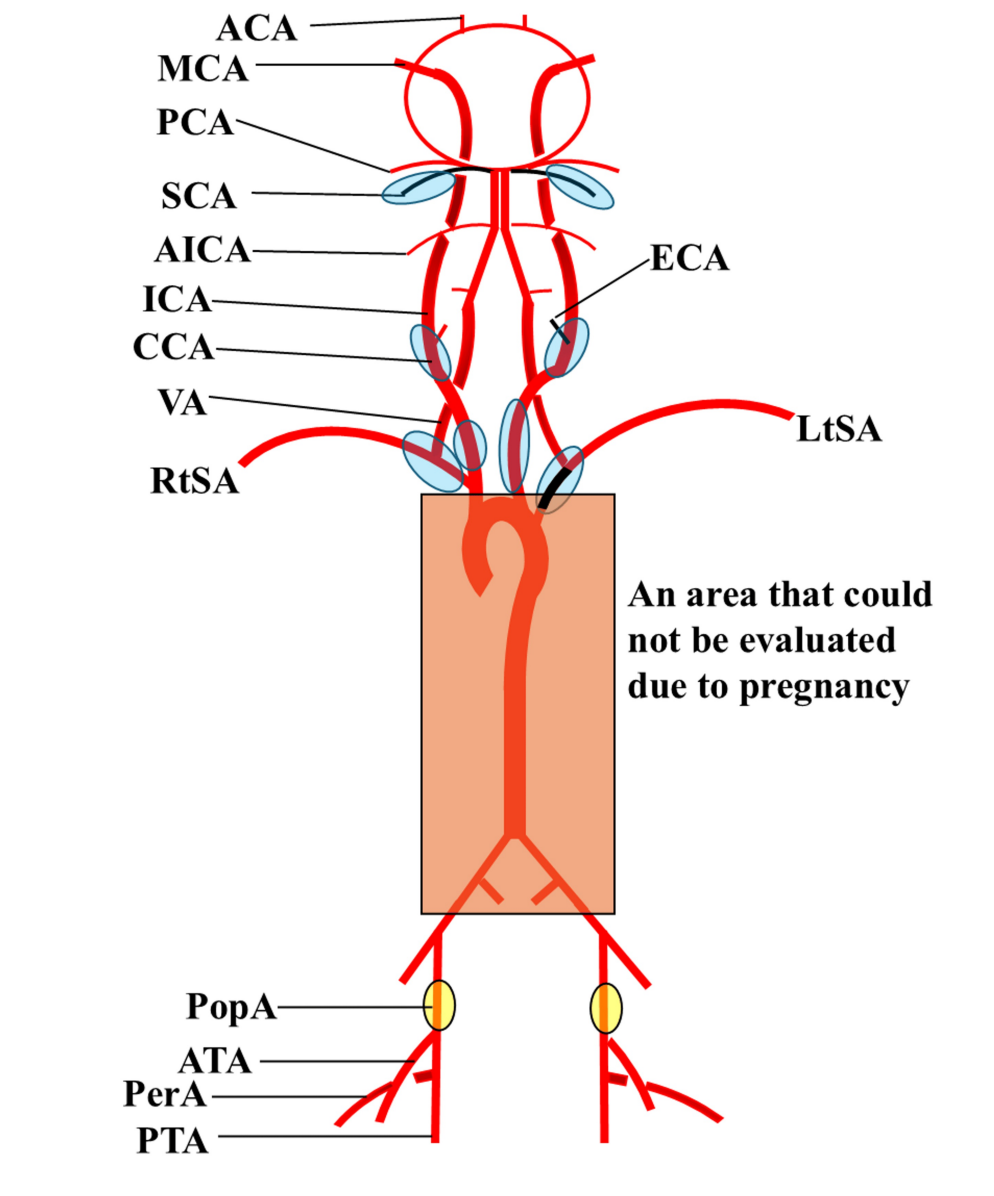
Overview of the patient's vascular lesions Regions outlined in blue represent stenotic lesions identified on contrast-enhanced CT performed 10 years prior. Regions outlined in yellow indicate stenosis suggested by current ultrasonography. Regions outlined in orange were not evaluated via imaging during the current pregnancy. Vessels indicated in black are occluded. ACA, anterior cerebral artery; AICA, anterior inferior cerebellar artery; ATA, anterior tibial artery; CCA, common carotid artery; ECA, external carotid artery; ICA, internal carotid artery; LtSA, left subclavian artery; MCA, middle cerebral artery; PCA, posterior cerebral artery; PerA, peroneal artery; PopA, popliteal artery; PTA, posterior tibial artery; RtSA, right subclavian artery; SCA, superior cerebellar artery; VA, vertebral artery Image credits: Created by the authors using Microsoft PowerPoint (Microsoft Corporation, Redmond, WA, US).

**Figure 2:**
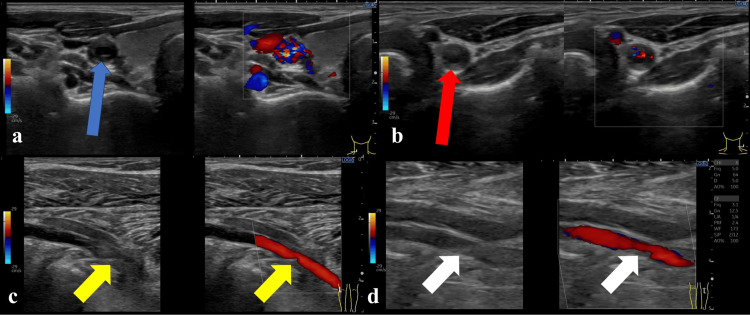
Ultrasonographic findings of the carotid and popliteal arteries a: Ultrasonographic image of the right CCA. The "macaroni sign," characterized by diffuse and circumferential vessel wall thickening, is clearly observed (blue arrow). b: Ultrasonographic image of the left CCA. Similar to the right side, the "macaroni sign" (circumferential vessel wall thickening) is observed (red arrow). c: Ultrasonographic image of the right popliteal artery. Significant vessel wall thickening and luminal narrowing are observed (yellow arrow), suggesting peripheral arterial involvement and potential disease progression. d: Ultrasonographic image of the left popliteal artery. Similar to the right side, wall thickening and narrowing are observed (white arrow), further supporting the suspicion of disease progression toward Numano type V [6]. CCA, common carotid artery

A 39-year-old pregnant woman (147 cm, 52.2 kg) at 35 weeks of gestation with an 11-year history of TA was referred for delivery management. Ten years ago, the patient presented to our hospital with primary complaints of dizziness, visual field defects, and speech impairment. Comprehensive evaluation revealed a cerebral infarction in the left parietal lobe. Notably, as the radial arteries were impalpable bilaterally, systemic vascular disease was suspected. Contrast-enhanced computed tomography showed extensive involvement (Numano Type I), including bilateral CCA stenosis and left subclavian artery occlusion (Figure 1) [6]. Current ultrasound revealed “macaroni sign” wall thickening in both CCAs and new involvement of both popliteal arteries (Figure 2). While a complete CT evaluation was deferred due to pregnancy, the new involvement of the popliteal arteries, combined with previous carotid findings, led to a presumptive classification of Numano Type V, reflecting likely progression from the initial Type I diagnosis 10 years prior. (Table 1) [6]. Notably, while ultrasound detected no obvious stenosis in the distal upper-limb arteries, systolic blood pressure was similarly low in both arms at approximately 60 mmHg (diastolic unmeasurable), compared to a lower-limb blood pressure of 120/60 mmHg. This discrepancy indicated severe proximal stenosis, consistent with the previously documented subclavian involvement, which remained undetectable by distal ultrasound, rendering upper-limb readings unreliable. Preoperative transthoracic echocardiography showed normal cardiac function with no wall motion abnormalities. Although coronary involvement was not reassessed via angiography due to the emergency setting and fetal radiation concerns, no history of coronary lesions existed in her previous records. Preoperative laboratory investigations revealed mild anemia (hemoglobin: 11.0 g/dL), which was considered physiological for the gestational age. Liver enzymes and platelet counts were within normal limits (aspartate aminotransferase: 11 U/L; alanine aminotransferase: 6 U/L; and platelets: 231 × 10^9^/L). Renal function demonstrated an increased estimated glomerular filtration rate (eGFR: 137 mL/min/1.73 m^2^), consistent with the typical physiological changes of pregnancy (Table 2). This elevated eGFR suggested the absence of significant renal artery stenosis, which can be associated with TA.

**Table 1:** Vascular ultrasound findings Reference ranges are specific to Seirei Hamamatsu General Hospital (Hamamatsu, Japan). Both CCAs exhibit the "macaroni sign," consistent with Takayasu arteritis. The right CCA shows increased peak systolic velocity, suggesting severe stenosis, whereas the left internal carotid artery was difficult to identify and suspected to be occluded. Due to diffuse arterial lesions and collateral circulation associated with Takayasu arteritis, blood flow velocities at each site should be interpreted considering systemic hemodynamic factors. ATA, anterior tibial artery; CCA, common carotid artery; EDV, end-diastolic velocity; ICA, internal carotid artery; ND, not detectable due to vascular occlusion; NE, not established; PopA, popliteal artery; PSV, peak systolic velocity; PTA, posterior tibial artery; VA, vertebral artery

		PSV (cm/s)	Reference values	EDV (cm/s)	Reference values
CCA	Right	290.7	40-100	127.8	5-30
	Left	61.4	40-100	18.8	5-30
ICA	Right	111.2	40-80	70.5	20-40
	Left	ND	40-80	ND	20-40
VA	Right	64.5	40-70	37.9	6-40
	Left	33.7	40-70	27.6	6-40
Pop	Right	60.4	54-82	7.3	NE
	Left	68.5	54-82	12	NE
ATA	Right	53.3	45-65	4.3	NE
	Left	43	45-65	3.8	NE
PTA	Right	65.7	45-65	0	NE
	Left	56.8	45-65	5	NE

**Table 2:** Preoperative laboratory findings Reference ranges are specific to Seirei Hamamatsu General Hospital (Hamamatsu, Japan). ALP, alkaline phosphatase; ALT, alanine aminotransferase; APTT, activated partial thromboplastin time; AST, aspartate aminotransferase; CRP, C-reactive protein; eGFR, estimated glomerular filtration rate; LDH, lactate dehydrogenase; PT-INR, prothrombin time-international normalized ratio

Blood test items	Values	Reference range
Sodium	139 mmol/L	138-145 mmol/L
Potassium	3.6 mmol/L	3.6-4.8 mmol/L
Chloride	106 mmol/L	101-108 mmol/L
Total protein	5.8 g/dL	6.6-8.1 g/dL
Total bilirubin	0.4 mg/dL	0.4-1.5 mg/dL
AST	11 U/L	13-30 U/L
ALT	6 U/L	7-23 U/L
LDH	142 U/L	124-222 U/L
ALP	110 U/L	38-113 U/L
CRP	0.17 mg/dL	0.00-0.14 mg/dL
Creatine kinase	28 U/L	41-153 U/L
Blood urea nitrogen	8 mg/dL	8-20 mg/dL
Creatinine	0.40 mg/dL	0.46-0.79 mg/dL
eGFR	137 mL/min/1.73m^2^	>60 mL/min/1.73m^2^
White blood cell count	8.1×10^3^/μL	(3.3-8.6)×10^3^/μL
Hemoglobin	11.0 g/dL	11.6-14.8 g/dL
Hematocrit	34.20%	35.1-44.4%
Platelet count	231×10^9^/L	(158-348)×10^9^/L
PT-INR	0.92	0.85-1.10
APTT	26.1 s	24-34 s
Fibrinogen	457 mg/dL	200-400 mg/dL

Emergency delivery was initiated after labor commenced prematurely. Upon gynecological examination, the cervix was fully dilated, and vaginal delivery was initially attempted. However, twenty minutes later, due to the failure of fetal descent and concerns regarding the patient’s limited physiological reserve to tolerate prolonged labor or potential postpartum hemorrhage, we prioritized maternal safety and proceeded with an emergency cesarean section. Due to the unreliability of upper-limb blood pressure and the need for continuous monitoring to prevent cerebral hypoperfusion during induction, initial attempts to secure a right dorsalis pedis arterial line were impossible due to aggressive body movements during contractions; placement was deferred until after anesthesia induction. Considering that lower-limb blood pressure might overestimate cerebral perfusion pressure due to bilateral CCA stenosis, we monitored rSO₂ (NIRO-200NX, Hamamatsu Photonics, Hamamatsu, Japan) as a surrogate marker of cerebral perfusion, as it provides valuable real-time information regarding cerebral oxygenation trends even when peripheral blood pressure measurements are unreliable. rSO₂ monitoring was initiated upon the anesthesiology team’s arrival in the operating room following the decision for an emergency cesarean section. No rSO₂ data were collected during the preceding stages of labor, as the patient was managed under standard obstetric monitoring prior to the surgical intervention.

**Figure 3:**
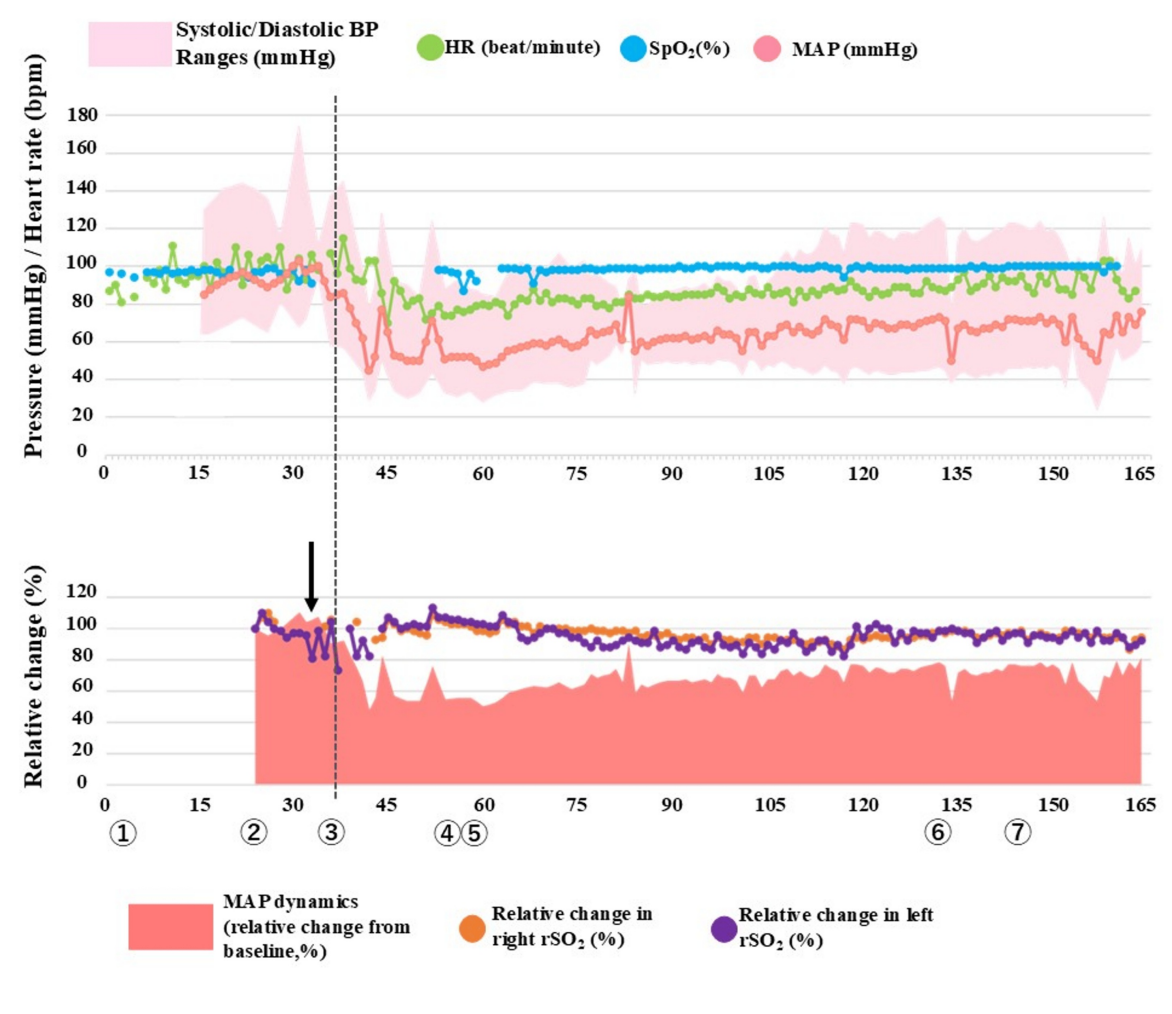
Intraoperative hemodynamic trends and rSO2 The upper panel displays the absolute values of mean arterial pressure (mmHg), heart rate (bpm), and SpO_2_(%). The lower panel shows the percentage change from baseline for rSO_2_. For both panels, the x-axis represents the elapsed time (minutes) from operating room entry. Markers: ① full cervical dilation and attempt at vaginal delivery; ② decision for cesarean section and commencement of anesthetic intervention; ③ administration of spinal anesthesia; ④ start of surgery; ⑤ delivery of the infant; ⑥ onset of headache; ⑦ end of surgery. Arrow: Immediately after positioning the patient in the lateral decubitus position for combined spinal-epidural anesthesia, rSO_2_ readings became unstable due to sensor detachment caused by profuse sweating and vigorous body movements during labor. Relative changes in rSO_2_ and MAP were calculated using values at point ② as the baseline. Following the administration of spinal anesthesia, both blood pressure and rSO_2_ showed consistent and correlated decreases. For details regarding the specific anesthetic agents and dosages administered, please refer to Table 3. MAP, mean arterial pressure; HR, heart rate; BP, blood pressure; rSO_2_, regional cerebral oxygen saturation

Combined spinal-epidural anesthesia was performed at the second to third lumbar interspace (10 mg hyperbaric bupivacaine and 20 μg fentanyl), achieving a sensory block to the C8 dermatome. In this case, combined spinal-epidural anesthesia was chosen over general anesthesia or graded epidural anesthesia to ensure a rapid onset of surgical block due to the emergency setting, while maintaining the ability to monitor the patient's consciousness. Although the sensory block reached C8, exceeding the standard T4 requirement, real-time rSO₂ monitoring allowed for precise hemodynamic management to prevent cerebral malperfusion despite the high block level. Post-induction, a right dorsalis pedis arterial line was secured. As blood pressure and rSO₂ decreased simultaneously, we prioritized rSO₂ as the primary indicator of cerebral perfusion pressure, using a 20% decrease from baseline as the threshold for vasopressors. Hemodynamics were managed with ephedrine, phenylephrine, and continuous norepinephrine, ensuring rSO₂ remained within 70% of baseline (Figure 3; Table 3). Mean arterial pressure ranged from 45 mmHg to 86 mmHg. Notably, decreases in mean arterial pressure were accompanied by corresponding reductions in rSO₂, allowing for early vasopressor intervention before sustained cerebral desaturation occurred. This synchronized trend confirmed the utility of rSO₂ as a primary surrogate for cerebral perfusion pressure in this patient. The patient reported a transient, non-positional headache 75 minutes into surgery, which resolved spontaneously without neurological deficits. After meticulous surgical hemostasis of the myometrium, the procedure was completed without any intraoperative complications. Total intraoperative fluid management included 2757 mL of infusion, with an estimated blood loss of 650 mL and a urine output of 625 mL. Both mother and neonate were discharged on postoperative day six without sequelae.

**Table 3:** Timeline of intraoperative clinical interventions and pharmacological management Timing indicates the specific time of administration, representing the elapsed time (minutes) since operating room entry. For medications administered multiple times, each individual timestamp is provided. These time points correspond to the elapsed time shown in the hemodynamic chart in Figure 3. Oxytocin (total 30 units) was administered via continuous intravenous infusion (10 units per 500 mL of acetated Ringer’s solution over 40 minutes, repeated three times) without any bolus doses.

Intervention/drug	Timing (min)	Dose/rate	Route
0.5% hyperbaric bupivacaine	36	10 mg	Spinal
Fentanyl	36	20 μg	Spinal
Ephedrine	40	4 mg	IV bolus
Phenylephrine	42, 47, 56, 62	0.1 mg	IV bolus
Norepinephrine	64-130	0.02-0.05 μg/kg/min	IV infusion
Oxytocin	60-180	30 units	IV infusion
1% mepivacaine	115	20 mg	Epidural
Fentanyl	115	50 μg	Epidural
Morphine	115	3 mg	Epidural

## Discussion

Challenges in hemodynamic monitoring

The primary challenge in managing this case was the absence of a reliable indicator for cerebral perfusion pressure. Preoperative imaging and the marked discrepancy between upper and lower limb blood pressure suggested extensive stenosis of the aortic arch branches. In TA patients with severe carotid artery stenosis, a divergence between peripheral blood pressure and actual cerebral perfusion is unavoidable. Given this patient's history of multiple cerebral infarctions, her cerebral perfusion reserve was deemed compromised, making her highly vulnerable to the rapid blood pressure decline typically induced by spinal anesthesia. While the patient's level of consciousness is a traditional indicator of cerebral perfusion, it is a late-stage consequence of circulatory failure and cannot serve as a proactive monitor to prevent ischemia. Although securing a superficial temporal artery line was considered, we prioritized rSO_2_ monitoring due to the technical difficulty of arterial cannulation during an emergency without sedation, and the potential unreliability of temporal artery pressure in the presence of external carotid artery lesions. This non-invasive method allowed for real-time assessment of the cerebral oxygen supply-demand balance, which was particularly advantageous in an emergency setting where invasive monitoring was difficult to establish.

Physiological validity of regional cerebral oxygen saturation ​​​​

A decrease in rSO₂ typically results from reduced oxygen supply, increased oxygen consumption, or venous congestion. In this case, the rSO₂ decline synchronized with the decrease in arterial blood pressure without a significant increase in deoxygenated hemoglobin. This suggests that rSO₂ sensitively reflected changes in oxygen supply, specifically cerebral blood flow (Figure 3). While the utility of rSO₂ for guiding anesthetic interventions remains a subject of debate [12-14], this controversy often pertains to standard patients where cerebral perfusion pressure can be adequately approximated via limb blood pressure. In specialized scenarios such as this, where conventional monitors fail to reflect cerebral hemodynamics-rSO₂ serves as a crucial "compass" for indirect assessment of cerebral blood flow. Although a definitive rSO₂ threshold for intervention in TA remains unclear, we initiated vasopressors at a 20% reduction from baseline. This 20% threshold is consistent with established clinical recommendations for managing cerebral oxygen desaturation to mitigate the risk of perioperative neurological complications [15].

rSO₂ reflects the balance between cerebral oxygen delivery and consumption and therefore serves as a surrogate marker of cerebral perfusion. In patients with extensive proximal arterial stenosis, peripheral blood pressure measurements may not accurately represent cerebral perfusion pressure. Under such circumstances, rSO₂ monitoring may provide valuable real-time information regarding cerebral oxygenation trends, although it does not directly measure cerebral blood flow or perfusion pressure. It should be noted that rSO₂ monitoring has inherent limitations; for instance, measurements may be influenced by extracranial blood flow and do not always provide a complete picture of global cerebral perfusion. However, by focusing on the percentage change from the patient's own baseline, we aimed to utilize rSO₂ as a practical trend monitor to guide hemodynamic intervention in this high-risk scenario.

Lessons from the emergency case

Recent reports indicate that a 5% decrease in rSO_2_ during elective cesarean section can predict maternal hypotension [16], leading to its increased use in obstetric anesthesia. Lee et al. reported successful management of an elective cesarean section in a TA patient where rSO_2_ values correlated with the onset and resolution of neurological symptoms [11]. While Lee et al. reported the utility of rSO₂ in an elective cesarean section [11], our case is unique in its focus on emergency management during active labor. Unlike previous reports where rSO₂ was used primarily as a supplementary tool, we utilized rSO₂ as the primary threshold for hemodynamic intervention. This suggests the reliability of cerebral oximetry as a leading indicator of cerebral perfusion in TA patients, particularly in emergency scenarios where peripheral blood pressure measurements are fundamentally unreliable.

In cases with such complex vascular pathology, anesthesia should ideally be initiated only after establishing comprehensive monitoring, including arterial lines and rSO_2_. However, in emergency situations where time is limited and sufficient preparation is not feasible, clinicians must be prepared to proceed with alternative monitoring strategies. In this case, we identified the following practical measures to overcome these limitations: 1) Reinforcing sensor fixation with additional adhesive tape to prevent detachment due to diaphoresis. 2) Assigning dedicated staff (e.g., a clinical engineer) to continuously monitor sensor contact. 3) Establishing a baseline trend for the patient’s upper limb blood pressure as a fallback, even if the absolute values are low, for situations where a lower-limb arterial line cannot be secured.

## Conclusions

In summary, rSO₂ served as a valuable additional indicator for managing a patient with TA and severely compromised cerebral perfusion. The dynamic nature of emergency labor can hinder the establishment of planned monitoring; thus, this case illustrates the need for clinicians to remain adaptable when facing physical and clinical constraints. While a single case report cannot define a new standard of care, our experience suggests that rSO₂ monitoring may be a helpful adjunct for real-time hemodynamic titration in complex obstetric cases. Further investigation remains necessary to establish standardized monitoring protocols for this high-risk population.

## References

[1] Saadoun D, Vautier M, Cacoub P (2021). Medium- and Large-Vessel vasculitis. Circulation.

[2] Toshihiko N (1996). Current status of large and small vessel vasculitis in Japan. Int J Cardiol.

[3] Comarmond C, Biard L, Lambert M (2017). Long-term outcomes and prognostic factors of complications in Takayasu arteritis: a multicenter study of 318 patients. Circulation.

[4] Grayson PC, Maksimowicz-McKinnon K, Clark TM (2012). Distribution of arterial lesions in Takayasu's arteritis and giant cell arteritis. Ann Rheum Dis.

[5] De Boysson H, Liozon E, Espitia O (2019). Different patterns and specific outcomes of large-vessel involvements in giant cell arteritis. J Autoimmun.

[6] Hata A, Noda M, Moriwaki R, Numano F (1996). Angiographic findings of Takayasu arteritis: new classification. Int J Cardiol.

[7] Pagnoux C, Mahendira D, Laskin CA (2013). Fertility and pregnancy in vasculitis. Best Pract Res Clin Rheumatol.

[8] Padiyar S, Manikuppam P, Kabeerdoss J, Rathore S, Danda D (2021). Update on pregnancy in Takayasu arteritis-a narrative review. Int J Rheum Dis.

[9] Taghiyeva A, Kılıç L, Cagan M (2023). Fertility, early menopause and pregnancy outcomes of patients with Takayasu's arteritis. Eur J Obstet Gynecol Reprod Biol.

[10] Comarmond C, Mirault T, Biard L (2015). Takayasu arteritis and pregnancy. Arthritis Rheumatol.

[11] Lee EH, Choi E, Ahn W (2013). Application of cerebral oximetry for a parturient with Takayasu's arteritis undergoing cesarean section -a case report. Korean J Anesthesiol.

[12] Moerman A, De Hert S (2015). Cerebral oximetry: the standard monitor of the future?. Curr Opin Anaesthesiol.

[13] Zorrilla-Vaca A, Healy R, Grant MC, Joshi B, Rivera-Lara L, Brown C, Mirski MA (2018). Intraoperative cerebral oximetry-based management for optimizing perioperative outcomes: a meta-analysis of randomized controlled trials. Can J Anaesth.

[14] Qiu L, Ma Y, Ge L, Zhou H, Jia W (2025). Efficacy of cerebral oxygen saturation monitoring for perioperative neurocognitive disorder in adult noncardiac surgical patients: a systematic review and meta-analysis of randomized controlled trials. World Neurosurg.

[15] Ortega-Loubon C, Herrera-Gómez F, Bernuy-Guevara C (2019). Near-infrared spectroscopy monitoring in cardiac and noncardiac surgery: pairwise and network meta-analyses. J Clin Med.

[16] Berlac PA, Rasmussen YH (2005). Per-operative cerebral near-infrared spectroscopy (NIRS) predicts maternal hypotension during elective caesarean delivery in spinal anaesthesia. Int J Obstet Anesth.

